# Getting the Lead Out: Biomolecular Crystals as Low-Cost, High-Performance Piezoelectric Components

**DOI:** 10.1021/accountsmr.2c00124

**Published:** 2022-07-20

**Authors:** Sarah Guerin

**Affiliations:** † SSPC, The Science Foundation Ireland Research Centre for Pharmaceuticals, University of Limerick, Limerick V94 T9PX, Ireland; ‡ Department of Physics, Bernal Institute, University of Limerick, Limerick,V94 T9PX, Ireland

## Introduction

**Figure 1:**
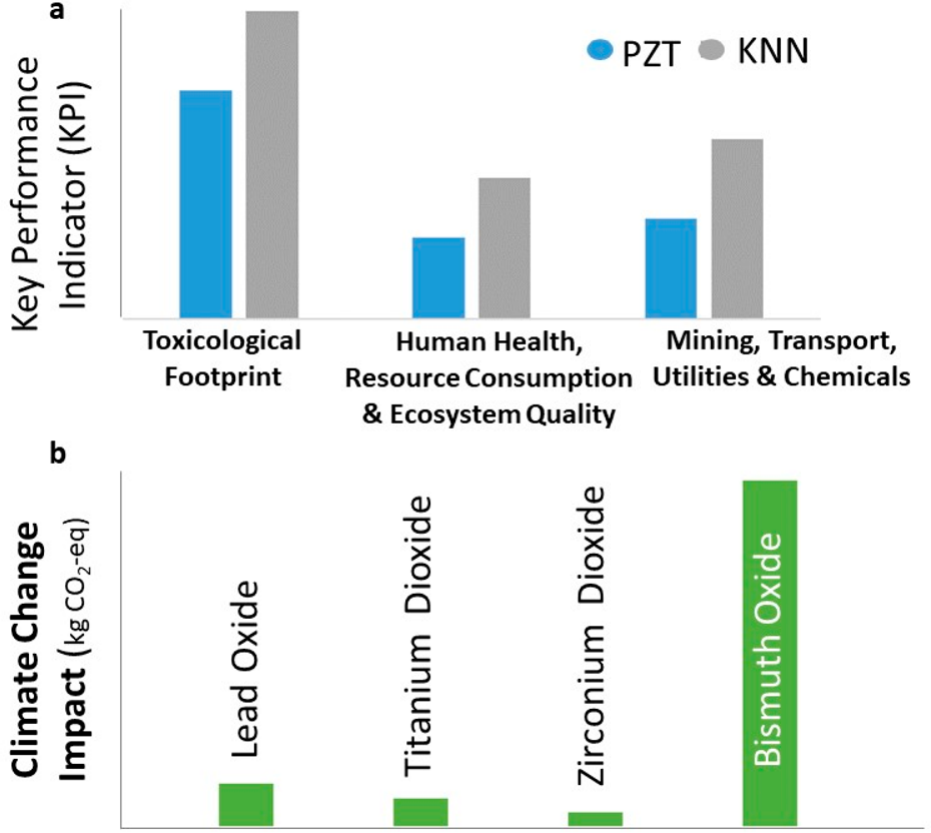
Piezoelectric ceramics are hugely damaging to the environment because of their required oxides.^1,2^

There are billions of piezoelectric sensors globally in our vehicles, consumer electronics, medical devices, advanced scientific equipment, fuel gauges, and structural health monitoring units. The vast majority of these sensors contain the perovskite lead zirconium titanate (PZT). It is estimated that there is 100 g of PZT distributed across a variety of integrated sensors in every one of the 1.4 billion cars on our roads. PZT requires toxic lead oxide (PbO) during its synthesis and leaches lead into water supplies at end-of-life disposal. Lead-free alternatives are a large field of research, yet the most-touted candidates, which are also ceramic materials containing elements such as niobium, bismuth, and barium, are even more damaging to the environment (Figure 1).

There are many obstacles to replacing existing materials in established technologies. From a scientific standpoint, key figures of merit (FoMs) need to be matched or exceeded and need to be reliable and reproducible over a required temperature range and within device lifetimes. The material and its processing should not be more complex or costly than its predecessor, and the environmental footprint should be a strong deciding factor in the coming decade as we face the devastating effects of human-made environmental damage. No current lead-free alternative meets all of these criteria, with the worst offenders being the most favored candidates: potassium sodium niobate (KNN), bismuth sodium titanate (NBT), and bismuth potassium titanate (KBT) ceramics. In the case of KNN, niobium pentoxide (Nb_2_O_5_) has the highest carbon footprint of all oxide raw materials, far exceeding PZT in its toxicological imprint, damage to ecosystems and human health, and consumption of resources^2^ (Figure 1a). As regards NBT and KBT, bismuth and its oxide (Bi_2_O_3_) are mainly the byproduct of lead smelting and Bi_2_O_3_ exceeds the environmental impact of PbO in several areas, including climate change (Figure 1b), because of additional processing and refining steps that pose extra challenges in metallurgical recovery. Bismuth also compares unfavorably with lead because of its higher energy cost of recycling. By contrast, biomolecular crystals (spanning amino acids, peptides, proteins, and viruses) have recently emerged as an exciting candidate for green piezoelectrics.^3^ Amino acid and peptide crystals significantly outperform PZT and its competitors in terms of cost, ease-of-processing, eco-friendliness, mechanical strength, and normalized voltage output. Over the next decade, it is hoped that targeted research will advance these truly green alternatives by laying the scientific groundwork for biomolecular piezoelectric materials to become disruptive, and truly environmentally friendly sensors.

## Biomolecular Crystal Technology: A Brief Overview

The discoveries that biomolecular crystals can exhibit a piezoelectric response on the order of 200 pC/N and can assemble as polycrystalline films represented key milestones in the development of biomolecular crystal assemblies as solid state actuators.^4^ The shear piezoelectric response in beta-glycine is the first to approach the longitudinal performance of commercial PZT (350–550 pC/N). Biomolecular crystals also exhibit piezoelectric voltage constants 1–2 orders of magnitude above PZT because of their low permittivity, despite it taking until the 21st century for a response above 1 pC/N to be reported in biomolecule assemblies. A key material in this renaissance has been the peptide diphenylalanine (FF), which assembles into unique micro- and nanostructures with promising piezoelectric, thermal, and mechanical properties. However, the maximum piezoelectric response in an FF-based material remains at 80 pC/N.^5^ The recent upsurge in the number of biological materials with piezoelectric constants between 10 and 100 pC/N is significant and demonstrates that biomolecular crystals are viable replacements for piezoelectrics such as aluminum nitride (AlN) and zinc oxide (ZnO). However, it is unlikely that the current approach will lead to the discovery of a material with a response of over 300 pC/N without intensive high-throughput screening of small biomolecular crystals. By generating large amounts of piezoelectricity data, and hence deciphering the key features of a piezoelectric crystal, researchers can target both high-performing molecular candidates and favorable growth environments to engineer superpiezoelectric crystal structures.

## So What Needs To Be Done?

Highly predictive density functional theory (DFT) models have created a platform to explore the origin of biomolecular crystal piezoelectricity, which can include a large net dipole in the single crystal unit cell, a large monoclinic angle (which corresponds to low shear stiffness), hydroxylated side-chain H-bond networks, and dense aromatic π–π zippers.^6^ A key limitation on progress has been the case-by-case nature of the investigations, which slows the generation of broadly applicable design rules. An ambitious computational workflow is required for nanoscale design of superpiezoelectric crystalline assemblies, ideally by combining high-throughput quantum mechanical calculations with machine learning algorithms. These computational endeavors should be integrated with the engineering of watertight polycrystalline assemblies with high thermal and mechanical stability, via controlled growth and systematic characterization. Innovative growth and standardized methodologies needed to enhance the piezoelectric response of crystal films include layered and molded deposition, thin polymer coatings to increase stability and insulate from water and contaminants, lightweight electrical contact to maximize resonant properties, and extensive evaluation of mechanical and thermal stability. Although proof-of-concept polycrystalline components can be developed with simple amino acids, the confluence in the field will be the feedback loop between the computational and experimental investigations to maximize piezoelectric performance beyond current technologies.

## Outlook

**Figure 2:**
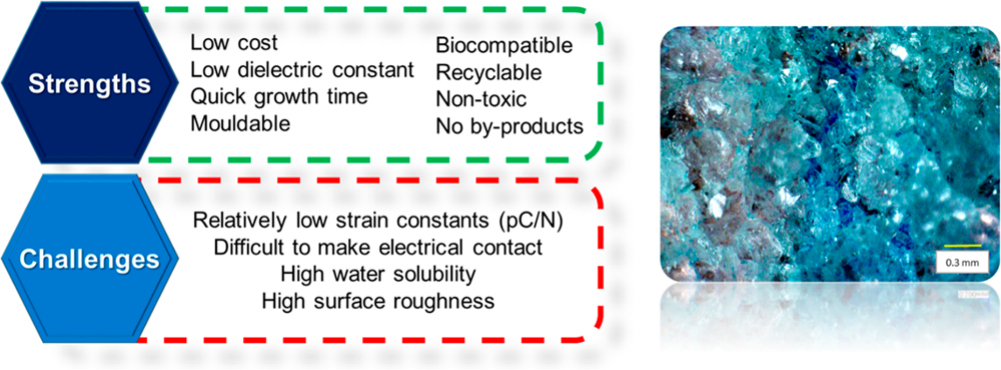
Some of the many strengths of biomolecular polycrystalline films, and the challenges that need to be overcome. An optical micrograph of a glycine polycrystalline film surface is shown on the right.

It is not possible in this short space to celebrate the diverse field of biomolecular crystal piezotechnologies. In the past 2 years alone, highly sensitive amino acid crystal actuators have been shown to be capable of ultralow pressure detection,^7^ and data-driven decision-making in identifying structurally degraded pipelines used in water distribution.^8^ Amino acid/polymer composites have been demonstrated to monitor wound healing^9^ and have been grown at the wafer-scale for biodegradable sensing.^10^ With targeted computational and engineering research in the coming years, the scientific and technical hurdles that are yet to be overcome for bio/organic piezoelectrics will become achievable challenges (Figure 2). If solved, this will produce a PZT-level piezoelectric response in eco-friendly materials with minimal batch-to-batch variability because of controlled polycrystalline growth. An optimiztic outlook sees us turn to in-silico crystal engineering, allowing us to sculpt and standardize the morphology of our polycrystalline device components to an unprecedented level, embedding desired electromechanical properties for successful applications in eco-friendly piezoelectric technology. Experimentally a shift is coming in the field toward methodologies that focus on maximizing the real-world device performance of biomolecular piezoelectrics, allowing for facile replacement of environmentally damaging alternatives.
